# Genetic structure in the Sherpa and neighboring Nepalese populations

**DOI:** 10.1186/s12864-016-3469-5

**Published:** 2017-01-19

**Authors:** Amy M. Cole, Sean Cox, Choongwon Jeong, Nayia Petousi, Dhana R. Aryal, Yunden Droma, Masayuki Hanaoka, Masao Ota, Nobumitsu Kobayashi, Paolo Gasparini, Hugh Montgomery, Peter Robbins, Anna Di Rienzo, Gianpiero L. Cavalleri

**Affiliations:** 1 Department of Molecular and Cellular Therapeutics, The Royal College of Surgeons in Ireland, Dublin, Ireland; 2 Centre for Human Health and Performance, and Institute for Sport, Exercise and Health, University College London, London, UK; 3 Department of Human Genetics, University of Chicago, Chicago, USA; 4 Department of Physiology, Anatomy and Genetics, University of Oxford, Oxford, UK; 5 Paropakar Maternity and Women’s Hospital, Thapathali, Kathmandu, Nepal; 6 First Department of Medicine, Shinshu University School of Medicine, Matsumoto, Japan; 7 Department of Legal Medicine, Shinshu University School of Medicine, Matsumoto, Japan; 8 University of Triests, Trieste, Italy; 9 Division of Experimental Genetics, Sidra, Doha, Qatar

**Keywords:** Nepal, Gene flow, principal component analysis, Admixture, Sherpa, Nepalese, Tibetan, Consanguinity, Subpopulations

## Abstract

**Background:**

We set out to describe the fine-scale population structure across the Eastern region of Nepal. To date there is relatively little known about the genetic structure of the Sherpa residing in Nepal and their genetic relationship with the Nepalese. We assembled dense genotype data from a total of 1245 individuals representing Nepal and a variety of different populations resident across the greater Himalayan region including Tibet, China, India, Pakistan, Kazakhstan, Uzbekistan, Tajikistan and Kirghizstan. We performed analysis of principal components, admixture and homozygosity.

**Results:**

We identified clear substructure across populations resident in the Himalayan arc, with genetic structure broadly mirroring geographical features of the region. Ethnic subgroups within Nepal show distinct genetic structure, on both admixture and principal component analysis. We detected differential proportions of ancestry from northern Himalayan populations across Nepalese subgroups, with the Nepalese Rai, Magar and Tamang carrying the greatest proportions of Tibetan ancestry.

**Conclusions:**

We show that populations dwelling on the Himalayan plateau have had a clear impact on the Northern Indian gene pool. We illustrate how the Sherpa are a remarkably isolated population, with little gene flow from surrounding Nepalese populations.

**Electronic supplementary material:**

The online version of this article (doi:10.1186/s12864-016-3469-5) contains supplementary material, which is available to authorized users.

## Background

The Himalaya was first colonised by modern humans approximately 25,000 years ago [1, 2]. Spanning Tibet, Nepal, India, Pakistan and Bhutan, the region is home to a vast number of ethnic groups residing at altitudes between 3,000 and 5,000 m above sea level.

Nepal is a “multi-ethnic” country, with 125 ethnic groups recorded in the 2011 Nepalese census [3, 4]. Reflecting this diversity, Nepal has a complex demographic history and has long served as a region of asylum due to its landlocked position between Asia and India. The first documented tribe in Nepal was the Kirats, a Tibeto-Burmese group that arrived in the region approximately 2,500 years ago [5]. Evidence suggest the Kirats first resided in Kathmandu but were forced to migrate to the high altitude terrain of the Khumbu valley, Eastern Nepal, around the 4th century following invasion by an Indian clan, the Licchavais [5, 6]. The Khumbu valley at the Tibet-Nepal border presents a challenging physical landscape and harsh environmental stresses to its residents. Despite this, it remains a well-populated region of Nepal and is native to the physiologically adapted ethnic group, the Sherpa. It is thought that the Sherpa migrated from the Salmo-Gang district of Kham, Eastern Tibet, to the Solu-Khumbu region of Eastern Nepal approximately 400–600 years ago due to political tension between Kham and their Northern neighbours- in Mongolia [7–9].

Previous studies have applied mitochondrial DNA (mtDNA) and Y-chromosome genetic systems as tools to reconstruct historic demographic events in Nepal. These studies have pointed to significant genetic structure across populations dwelling in Nepal. For example, a South-Central European origin has been attributed to Y chromosome haplotypes prevalent in the Nepalese Newar population, interpreted as suggesting gene flow from India into Nepal. In contrast, haplotypes observed in the Nepalese Tamang population are commonly observed in Tibetan populations, suggesting ancestry from the North of the Himalaya [10–12]. These distinct patterns have also been reproduced using mtDNA-based systems [13] and imply differing ancestral contributions from Tibet, India and bordering regions to contemporary Nepalese populations. Collectively, these results suggest considerable admixture in the Nepalese population.

The study of autosomal genetic variation appears to support the theory of admixture in Nepalese populations. A recent survey of multiple Nepalese and Burmese populations pointed to significant genetic differentiation between populations residing within the Himalaya. This differentiation appeared to be structured according to the principal linguistic phyla in the region-Tibeto-Burman and Indo-European, suggesting that both language and geography were influencing gene flow in the region [14]. However, the number of autosomal loci studied by the authors was limited relative to current approaches. Further, the origin of gene flow from south of the Himalaya to Nepal has not been described.

A recent study, using dense autosomal genotype data, explored the genetic history of Tibetans and Sherpa residing at high altitude [15]. The study identified a common ancestral component shared between these two high altitude populations, which was absent from lowland South or Central Asian populations. The proportion of this ‘high altitude’ ancestral component was highly enriched in the Sherpa while the Tibetans exhibited admixture of this, and an East Asian component enriched in the contemporary Han Chinese. A recent study using mtDNA and Y-chromosome markers also revealed strong affinities between the Sherpa and Tibetans [16]. The authors alluded to homogeneity of particular haplogroups within the Sherpa suggesting a founder effect from a small number of migrants from a Tibeto-Burman source population [16]. Despite in depth analysis of the genetic origins of the Sherpa, there has been no investigation into the genetic structure of the Sherpa in the context of their current residence in Nepal.

We set out to describe fine-scale population structure and admixture of the Sherpa and their neighbouring Nepalese populations using dense genomic datasets. We performed a detailed investigation of the genetic architecture of seven Sherpa villages located in the Khumbu region of Eastern Nepal and five Nepalese ethnic groups, which collectively represent the majority of the Nepalese population.

## Results

### Population substructure reflects geographical boundaries of the Himalaya

**Fig. 1:**
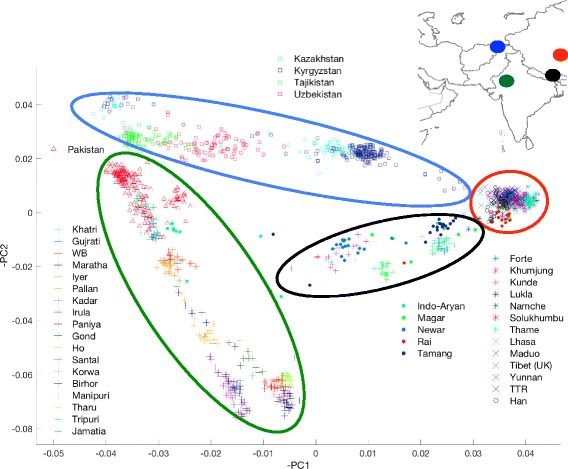
Genes mirror the geography of the greater Himalayan region. Legend Fig. 1. PC1 and PC2 explain genetic distance between populations as directions of variance. This was performed on a thinned dataset of 34,253 SNPs. Each dot represents an individual in the dataset. Each population is represented by a shape, Tibetans (X), Sherpa (★), Han (O), Nepalese (•), Indian (+), Pakistan (Δ) and the Pamir populations (□). Each of the Indian ancestral groups as described in Basu et al., 2016, are indicated as follows; [] ANI, + ASI, * AAA, ** ATB. The Nepalese appear as an admixed population between the northern Himalayan cluster (Tibet, Sherpa and Han) and the southern Himalayan cluster (India and Pakistan), with clear genetic variance between ethnic subgroups

We performed principal component analysis (PCA) (see methods), to provide a broad overview of population structure across the Himalaya (see Fig. 1). Four broad population clusters were apparent; 1) a ‘northern Himalayan’ cluster consisting of Han Chinese, Tibetans and the Sherpa, 2) A ‘northwestern’ cluster consisting of populations of the Pamir mountain range, 3) a ‘southwestern’ cluster consisting of Pakistani and Indian populations and 4) a ‘central Himalayan’ cluster consisting of the Nepalese.

The Nepalese as a whole appear as a potentially admixed population on the PCA, lying between the ‘southern’ (Indian) and ‘northern Himalayan’ (Tibetan) clusters. Interestingly, the distinct Nepalese ethnic subgroups (see Additional file 1: Table S2 for cohort details) would appear from PCA to have different proportions of gene flow from the ‘northern’ and ‘southern’ Himalayan regions. It is noteworthy from the PCA, that the Sherpa show, virtually zero genetic influence from southern populations, clustering with the Tibetans and the Han (see supp Fig. 1). These results are consistent with previous reports of gene flow in a north to south direction over the Himalayan barrier into Nepal, with limited gene flow in the opposite direction [12].

To investigate the extent of population substructure between the seven Nepalese Sherpa villages in the Khumbu valley, PCA was performed on Sherpa individuals only. Genetic substructure was evident across the Sherpa villages, with individuals from the village of Thame separating on PCA from members of other villages. The patterns observed from the PCA map well to the geography of the region (Additional file 2: Figure S2).

### Genetic distance and admixture proportions of Himalayan populations reflect the demographic history of the region

**Fig. 2:**
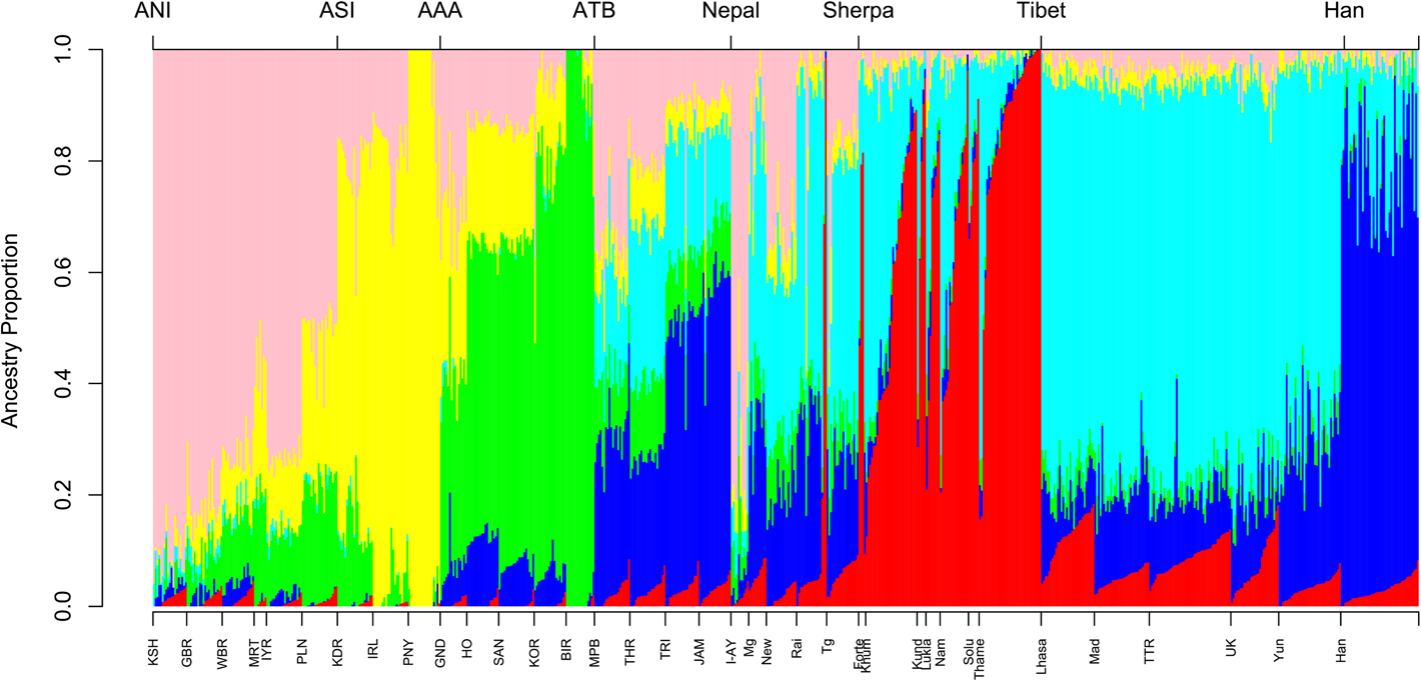
Fine scale analysis of the Nepalese and Sherpa. Legend Fig. 2. Admixture results for k = 6 were plotted for the best fit of the data. The x-axis labels each ethnic group or village (see Additional file 2: Table S2 for IDs). The labels above the plot indicate the main population groups; The Indian ancestral groups are defined as in Basu et al., 2016, ANI (ancestral north India), ASI (ancestral south India), AAA (ancestral Austro-Asiatic), ATB (ancestral Tibeto-Burman). The red component reflects the ‘ancestral high altitude component’ previously reported by Jeong, Alkorta-Aranburu (14). Our Nepalese cohort shows similar ancestral components to Indians of Tibeto-Burman ancestry (17). (36,330 SNPs)

To further our understanding of potential admixture events in the Nepalese subpopulations, we next conducted a model-based qualitative assessment of ancestry using the software ADMIXTURE [17]. Based on information provided by the PCA analysis, we restricted the ADMIXTURE analysis to the Sherpa, Nepalese, Tibetans, Han Chinese and Indians. Results indicate the Sherpa are a homogenous population, relative to the Nepalese who appear admixed for ‘northern’ and ‘southern’ Himalayan ancestry. At k = 6, the best fit of the data, we observed two ancestral components (red and cyan components in Fig. 2) specific to individuals from Tibet and Nepal, including the Sherpa. One of these components is enriched in the Sherpa (red) and found to be at higher proportions in Tibetans from Lhasa and the Tuo Tuo River. The second is enriched in Tibetans, but is also present in the Nepalese and Sherpa (cyan). We believe these two components broadly reflect the ‘ancestral high altitude component’ previously reported by C Jeong et al., [15]. We also note the Sherpa from Thame as being enriched for the red component, while the Sherpa from the remaining Nepalese villages show admixture of the two ancestral components specific to highlanders.

A recent study of Indian population structure described four ancestral components associated with contemporary mainland Indian populations [18]. Given the apparent Indian ancestry in the Nepalese populations studied here, we asked which of these four Indian ancestral components were most prevalent in the Nepalese populations. We began by identifying the four Indian ancestral components in the ADMIXTURE analysis, using the same dataset as the original report [18]. The vast majority of the Nepalese populations we studied associated most closely (on both PCA and ADMIXTURE) with the Tibeto-Burman Indians (ATB) located in Northeast India. The exceptions are the Sherpa (who have no significant Indian ancestry) and the Indo-Aryan, who show affinity with the Ancestral North India (ANI) group. We note that the ‘ancestral high altitude component’ is present within ATB ancestry (cyan in Fig. 2), indicating a significant influence on the Northern Indian genepool from ancestral populations dwelling on the Himalayan plateau.

We next calculated a 3-population F_st_ (F_3_) to quantify population differentiation across the Himalaya observed by PCA and admixture (Additional file 1: Table S3). Increased F_3_ values indicate recent shared ancestry between a pair of populations. F_3_ results correlate well with the patterns observed via PCA. The largest F_3_ values were observed between the Han and Tibetans, and the Sherpa and Tibetans, reflecting the recent common ancestry of these pairs of populations [15]. We performed a fine-scaled 3-population F_st_ between Indian and Nepalese ethnic groups (Additional file 1: Table S4). Interestingly the Nepalese in general appear closer genetically to the Tibetans than to Indian populations, suggesting strong Tibetan origins of at least some of the indigenous Nepalese ethnic groups. Weir and Cockerhams pairwise F_st_ was then calculated on a micro scale to measure genetic variance between the five Nepalese ethnicities and the Sherpa of the Khumbu valley. This identified the Rai, followed by the Magar and Tamang as the genetically closest Nepalese ethnic groups to the Sherpa (Additional file 1: Table S5). It is noteworthy that the Magar are believed to have originally migrated from the same region of Tibet as the Sherpa [19].

### Subpopulations of Nepal show various degrees of admixture

To determine which Indian ethnic subgroup was the strongest contributor to the ‘Southern’ component observed in the Nepalese population as a whole (the subgroups included in the ‘Nepalese’ population are defined in the methods, cohort section on Nepal), we performed a 3-population test using two source populations, Tibetans (representing Northern-Himalaya) and each of the four ancestral Indian ethnic groups (ANI, ASI, AAA and ATB) (representing ‘Southern Himalaya’). We identified significant signals of admixture (Z < −5) in the Nepalese for northern and southern Himalayan ancestry, the most significant admixture event being between the ANI and Tibetans (Additional file 1: Table S6). We did not detect a significant signal of admixture in the Sherpa, but we did confirm the well-established Han admixture in Tibetans.

**Fig. 3:**
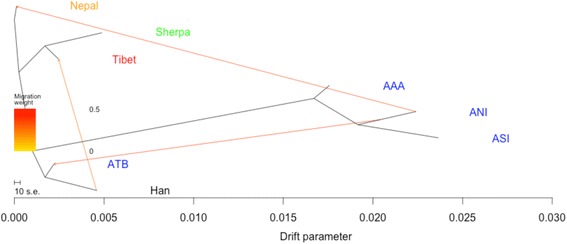
Modelling gene flow in Nepal. Legend Fig. 3. To infer patterns of population mixture in Nepal, we plotted a maximum likelihood tree using the software tool Treemix, allowing three migration events. Migration events are indicated by the arrows and coloured according to their weight corresponding to the coloured bar on the left. Populations from India are labelled in blue; ANI (ancestral north India), ASI (ancestral south India), AAA (ancestral Austro-Asiatic) and ATB (ancestral Tibeto-Burman)

To confirm this proposed admixture event in the broad Nepalese population we modeled a maximum likelihood tree between our populations of interest using the software tool Treemix. Results show the Nepalese, Sherpa and Tibetans to be closely related populations, with gene flow from ANI into the Nepalese population (Fig. 3).

We then applied the d-statistic test to inform on whether Tibetan ancestry in Nepalese populations was the result of gene flow from Tibet in to Nepal, or rather Nepal into Tibet. Results suggested much stronger gene flow from Tibet in to Nepal, rather than from Nepal in to Tibet (Additional file 1: Table S7).

Next, we quantified the proportions of admixture in the Nepalese ethnic groups using the F4-ratio estimation. We assigned the Tibetans and the ANI as ancestral source populations for the Nepalese, based on 3-population test results. The Newar appeared as the most admixed Nepalese ethnic group, with similar proportions of Tibetan and ANI ancestry (Table 1). The Rai, Magar and Tamang showed 92%, 82% and 79% Tibetan ancestry, respectively. The Indo-Aryan showed 93% ANI ancestry. These ancestral proportions are consistent with results from our PCA and Admixture analysis. These admixture proportions reported here across Nepalese subpopulations also concur with demographic literature of Nepal and previous mtDNA and Y-STR studies (see supp cohort description for Additional file 3) [10, 14].

**Table 1:** F4-ratio quantifying admixture in Nepalese ethnic groups

A	O	X	C	A	O	B	C	alpha	std.err	Z
Sherpa	Yoruba	Indo-Aryan	ANI	Sherpa	Yoruba	Tibet	ANI	0.073	0.009	7.839
Sherpa	Yoruba	Rai	ANI	Sherpa	Yoruba	Tibet	ANI	0.937	0.009	102.944
Sherpa	Yoruba	Tamang	ANI	Sherpa	Yoruba	Tibet	ANI	0.792	0.008	97.579
Sherpa	Yoruba	Magar	ANI	Sherpa	Yoruba	Tibet	ANI	0.872	0.011	82.170
Sherpa	Yoruba	Newar	ANI	Sherpa	Yoruba	Tibet	ANI	0.521	0.008	65.542

Legend Table 1. We used the F4 ratio to quantify the proportion of admixture in each of our Nepalese populations. Population X is being tested for admixture. Alpha is the proportion of population B ancestry in population X, while 1 – alpha is the proportion of population C ancestry in population X. See Additional file 2: Figure S3, a phylogeny, for further explanation

The (Tibetan-ANI) admixture events were then dated in the admixed Nepalese subgroups (Newar, Magar and Tamang) using Rolloff [20]. Results correlate well with the documented arrival dates of these ethnic groups to Nepal [6, 21]. Assuming a generation time of 30 years, our analysis dated an admixture event to have occurred in the Newar 1,504 YBP (years before present, 50.13 generations), which correlates well with historical records that have suggested the Newar have resided in Nepal since the early 4th century [21, 22]. We dated admixture events for the Tamang and Magar as 1233 YBP (41.09 generations) and 866 YBP (28.87 generations) respectively. These dates correlate well will historical records that suggest the Tamang’s arrival in Nepal around the 8th century, and the Magar’s in Nepal in the 12th century [5, 23].

### Patterns of homozygosity suggest recent consanguineous unions in a number of Sherpa and Nepalese subgroups

We investigated the extent of homozygosity in populations across the Himalaya to shed further light on the demographic history of the region. Elevated levels of runs of homozygosity (ROH) can be used to inform on isolation and consanguinity within a population [24, 25]. We measured ROH for a number of threshold lengths which can be used to infer the degree of shared parental ancestry, including ROH ≥ 1mb which are suggestive of ancient relatedness and ≥ 16mb (ROH16), which are suggestive of recent inbreeding [24].

**Fig. 4:**
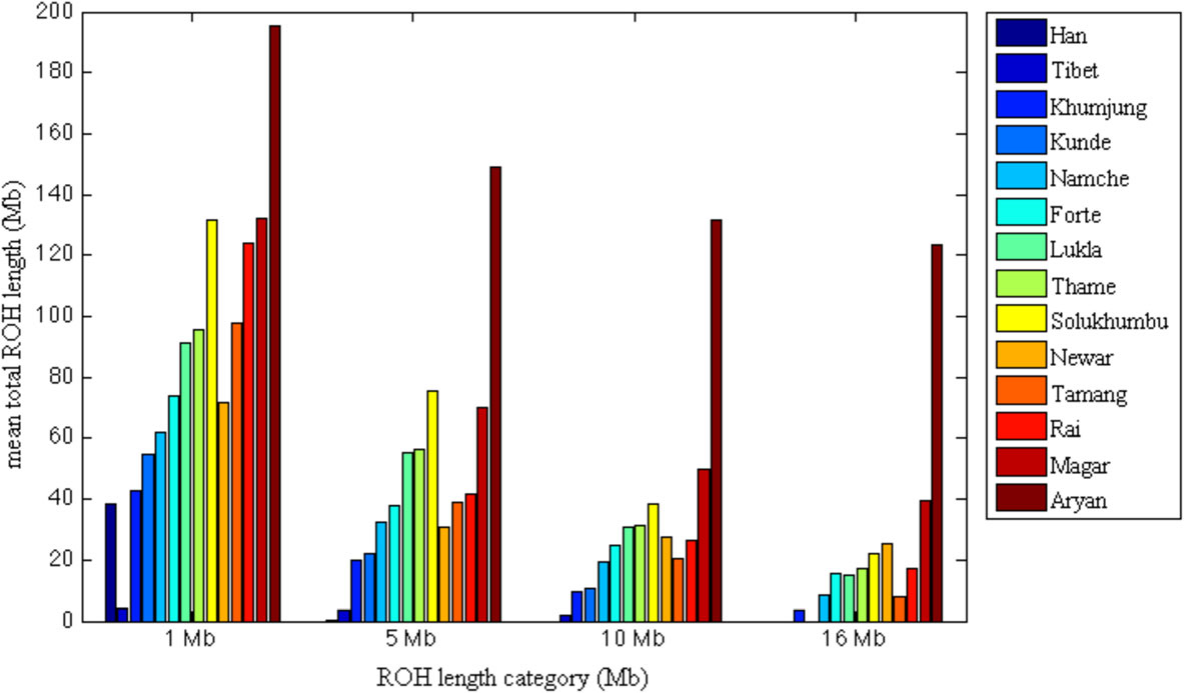
Levels of homozygosity in Sherpa and Nepalese populations. Legend Fig. 4. The x-axis represents the thresholds set for defining a ROH (≥1 Mb, ≥5 Mb, ≥10 Mb and ≥ 16 Mb). The y-axis is the mean total ROH length calculated for that population. We detected elevated proportions of ROH16 in the Nepalese Indo-Aryan, Magar, Newar, Rai, Tamang, and the Sherpa from Forte, Solukhumbu, Lukla and Thame

Notably elevated levels of ROH16 were also observed in the Sherpa and Nepal cohorts (see Additional file 2: Figure S4), despite the fact that consangiunous kinships are traditionally not permitted in these populations [26]. We next measured ROH for each of the Sherpa and Nepelase subpopulations independently to determine if particular groups were driving the elevated ROH signal (see Fig. 4). We observed considerable variability in homozygosity levels across subpopulations of the Sherpa and Nepalese. The Nepalese Indo-Aryan stand out as having the longest ROH detected across all thresholds tested. Elevated levels of ROH were also detected in all the other Nepalese groups (Magar, Rai, Newar and Tamang), and Sherpa from the villages of Thame, Lukla, Namche, Forte and Solukhumbu, suggesting both ancient and recent shared ancestry as a cause of elevated homozygosity in these subgroups. The observed patterns of homozygosity across the ROH thresholds for the Sherpa from Khumjung and Kunde indicates ancient shared parental ancestry, most likely due to isolation and small N_e_, with little recent consanguinity (indicated by negligible ROH16).

To test for consanguinity within the Nepalese and the Sherpa subgroups that showed elevated ROH16, we compared identity by descent (IBD) segments between pairs of individuals within a population, to ROH within individuals from the population. Where recent consanguinity is present, one would expect a significant increase in ROH within, compared to IBD between, members of that population. We set an IBD segment threshold of 16 Mb as indicative of a recent common shared ancestor between a pair of individuals. Results are illustrated in Additional file 2: Figure S5. We found ROH16 to be significantly greater than IBD16 for the Nepalese Indo-Aryan supporting consanguinity as an explanation for observed ROH patterns in that population, despite unions between biological kin being prohibited [27]. Although ROH16 was also greater than the IBD16 for all other Nepalese ethnic groups tested, the results were not significant. Despite the high levels of homozygosity previously detected in a number of the Sherpa subgroups, significant differences between IBD16 and ROH16 suggest this is an artifact of population isolation rather than the practice of consanguineous unions.

## Discussion

Our findings reveal that geography has influenced the shaping of genetic structure in the Himalaya at both a macro and micro level. The observation from PCA of four broad population clusters across the Himalaya is consistent with recent reports [13, 14, 28]. The micro-level influence of geography is apparent from the PCA results at the Sherpa village level. We confirm significant admixture in the Nepalese population as a whole, resulting from the mixing of populations from north and south of the Himalaya. We observed differential proportions of ancestry across the Nepalese subgroups we studied. We noted the Sherpa as remarkable in the Nepalese context, in that they have experienced little or no admixture with other Nepalese populations.

We have illustrated the presence of a Himalayan ancestry component in contemporary Indian populations, specifically those defined as Ancestral Tibeto-Burman (ATB) by Basu et al. [18]. It is interesting to note the large ‘East-Asian’ component in the ATB population, relative to the Himalayan component (blue vs. cyan in Fig. 2), suggesting the origin of the Himalayan component in ATB is via a more easterly route (Burma) rather than from Tibet. In any case it is clear that ancestral populations dwelling on the Himalayan plateau have had a clear impact on the Northern Indian gene pool.

Large mountainous regions can be expected to influence favourable directional gene flow between frontiers. Physiological stress imposed by high altitude may restrict gene flow from areas of low altitude to high altitude [12]. However, directional migration may also be an effect of economic factors or political instability. Results presented here would suggest a greater penetration of Himalayan ancestry into the North Indian gene pool rather than vice-versa. We confirmed high levels of gene flow into Nepal from north of the Himalayan watershed by D-statistic analysis, but not in the opposite direction.

Although it is well established that the Nepalese are a highly admixed population, the extensive structuring of admixture across subpopulations has not previously been described. We identified the Rai, Magar and Tamang to carry the greatest proportions of Tibetan ancestry, respectively. These three Nepalese ethnicities have been recognized to have Tibetan origins, and all speak Tibeto-Burman languages [8, 19, 29]. The Indo-Aryan, an Indo-European Nepalese ethnic group, strongly resemble the ANI populations given their large component of north Indian ancestry identified by admixture and the F4-ratio.

Despite the Sherpa’s residence in Nepal for the past 400–600 years, the Sherpa remain as an isolated homogenous population with little or no gene flow from their immediate Nepalese neighbours or any populations south of the Himalaya. The Sherpa genome is enriched for the previously reported ‘high altitude ancestral component’ [15] (Fig. 2), reflecting common ancestry with Tibetans and migration from Tibetan before the more recent movement of northern populations in to Tibet. As an isolated and relatively small community, drift will play a major role in shaping the Sherpa genome.

We detected considerable levels of autozygosity within the Sherpa and their neighboring Nepalese populations. The patterns of ROH we observed across the majority of Sherpa and Nepalese subpopulations indicate both ancient and recent parental relatedness. IBD analysis confirmed that the Nepalese Indo-Aryan appear to be the only significant consanguineous subgroup. We believe the elevated IBD16 in the Sherpa reflects a history of population isolation due to the remote location of high altitude villages in eastern Nepal and is also suggestive of founder effect [30]. This can be supported by recent findings identifying internal homogeneity for particular mtDNA and Y chromosome haplogroups during the origin of the Sherpa [16, 31, 32].

We note an important limitation of our study: that of the 125 recognized ethnic groups in Nepal [4], we only considered a subset. Thus, whilst our results inform on ethnic groups to which the majority of the population are members, we have studied a minority of the ethnic groups present in Nepal.

## Conclusion

In summary, analysis presented here illustrates the impact of broad Himalayan geography on genetic structure in the region. Significant gene flow from north of the Himalaya to Nepal is clearly evident, with relatively little gene flow in the opposite direction. Ethnic subgroups within Nepal show distinct genetic structure, reflecting differing histories of admixture and isolation. The Sherpa appear as a remarkably isolated population, with little gene flow from surrounding Nepalese populations.

## Methods

### Cohorts

#### Nepal

The 2011 Nepalese census recorded 125 distinct ethnic groups [4]. Using this census data and descriptors of the “*Nepal Federation of Indigenous Nationalities”,* we selected the most common indigenous Nepalese ethnic groups that collectively represent approximately 50% of the population of the Eastern Mountain and Hill Region of Nepal (Additional file 1: Table S1) [4, 33]. The four Nepalese populations selected based on census data were as follows; Rai (*n* = 20), Magar (*n* = 10), Tamang (*n* = 18) and Newar (*n* = 17). We also included the Indo-Aryan (*n* = 11) considering their close ethnic relation to the Newar [34]. For the purpose of downstream genetic analysis these Nepalese ethnic groups were merged to form the “Nepal” cohort. Nepalese ethnic groups were recruited from regions in close proximity to the Sherpa including Lukla, Solukhumbu and Kunde. For additional information on the Nepalese cohort see Additional file 3 [35–40].

#### Sherpa

We established a cohort of 118 Sherpa from three distinct recruitment efforts, including 49 previously genotyped individuals [15]. The Sherpa were sampled from seven high altitude villages in the Khumbu region of Eastern Nepal, namely Thame (*n* = 43), Khumjung (*n* = 30), Namche (*n* = 19), Lukla (*n* = 9), Khunde (*n* = 7), Forte (*n* = 3) and Solukhumbu (*n* = 7). Details of two of these recruitment efforts have been described previously [15, 41]. We will refer to these samples collectively as the “Sherpa” cohort.

For fine scale population analysis we have catagorised the Sherpa by village and the Nepalese by their ethnic group (see Additional file 1: Table S2).

#### Tibetans

We extended our cohort to include other populations representing the greater Himalayan region. We included formerly genotyped Tibetans from Lhasa (*n* = 29), Yunnan (*n* = 35), Tuo Tuo river (*n* = 46), Maduo (31), and Tibetans resident in the UK (*n* = 27) [42–45].

#### Pamir

Previously genotyped individuals representing populations along the Silk Road were also included, considering their cultural and economic ties with populations of Central Asia [46]. These consisted of individuals from Alga and Almaty in Kazakhstan (*n* = 59), Bukhara, Karshi, and Tashent in Uzbekistan (*n* = 83), Shing, Zeravshan, Kalaikhum, Khorog, and Rushan in Tajikistan (*n* = 83) and Krakoi and Kara-koo in Kirghizstan (*n* = 184). We refer to these populations collectively as the “Pamir” cohort.

#### Other Asian cohorts

We also included genotype data for 331 individuals from 18 mainland Indian populations kindly provided to us by Basu et al. [18] and individuals from the Human Genome Diversity Project to include the Han (Beijing China, *n* = 44), and individuals from Lahore (Pakistan, *n* = 168). See Additional file 1: Table S2 for detailed information of recruitment sites for all individuals included in the analysis.

### Isolation of white blood cells and DNA purification from buffy coat

DNA isolation and purification was completed for 36 Nepalese and 21 Sherpa (from Lukla, Solukhumbu and Kunde). Venous blood was collected from each subject in 2 ml EDTA vacuettes. White blood cells were isolated following a standard lysis protocol and DNA was purified using the QIAamp DNA mini kit, Qiagen.

### Genotyping

Genotyping was performed on 82 Nepalese, and 69 Sherpa individuals at the Wellcome Trust clinical research facility, Edinburgh. The Illumina OmniExpressExome BeadChip 8v1–2 system captured 964,193 SNPs.

### Genotype quality control

Quality control (QC) was performed in PLINK V1.07 [47] on each population individually before merging. Individuals and SNPs with genotype call rate of <95% were excluded. SNPs with a minor allele frequency <2% or with a Hardy-Weinberg *p* < 0.001 were excluded. Individuals in each population were checked for cryptic relatedness and where identity by descent (IBD) scores of >0.125 (3rd degree relative) were identified one from each such pair was removed. An exception to this IBD filter was made for the Sherpa where an IBD score <0.180 was accepted since the Sherpa seemed in general to have a higher degree of cryptic relatedness, possibly due to the isolation of the villages and small population numbers [48, 49]. Individual populations were then merged for analysis using only a common subset of SNPs, and QC was repeated using the same criteria.

For downstream analysis we refer here to ‘full’ or ‘thinned’ datasets. The ‘full’ dataset is that where all SNPs that passed standard QC were used, including the SNPs that are in high linkage disequilibrium. The ‘thinned’ dataset was prepared in PLINK, on the merged population datasets. Linkage disequilibrium was calculated (r^2^) between each pair of SNPs in a 1000 SNP window, and one of a pair of variants dropped from the dataset where LD (r^2^) between that pair was >0.8. The sliding window was then shifted 50 SNPs and the process repeated throughout the entire dataset.

The final, post-QC dataset consisted of 1245 individuals comprising of 103 Sherpa, 76 Nepalese (across five ethnicities; 17 Rai, 10 Magar, 18 Tamang, 17 Newar and 10 Indo-Aryan), 137 Tibetans, 44 Han, 168 Pakistan, 326 Indians (across 18 mainland Indian ethnic groups as described in Basu et al., [18], 59 Kazakhstani, 83 Uzbekistani, 83 Tajikistani and 184 Kirghizistani.

### Principal component analysis

Principal component analysis (PCA) was performed on the ‘thinned’ dataset using routines available via Genome-wide Complex Trait Analysis (GCTA) [50]. Results were plotted using Matlab R2011a.

### Admixture

Maximum likelihood estimation of individual ancestries was run using the software package ADMIXTURE [17]. Using our thinned dataset, values of K = 2–9 were run in replicates of 100, using different random seeds. K represents the number of ancestral components to be inferred. Cross-validation (CV) errors and log likelihood values were recorded for each replicate. The top ten log likelihood values do not differ >1 and the lowest CV error was chosen, for each K value and plotted, as the best estimation of ancestral fit.

### AdmixTools

To quantify admixture events between populations we applied the 3-population test, d-statistic, F4 ratio estimation and Rolloff following standard protocols as implemented in the software package AdmixTools (V. 3.0) [20]. All these analysis were performed on the ‘full’ dataset. We used the 3-population test (a generalisation of a 3-population F-statistic) to measure genetic variance between pairs of populations across the Himalaya, the Yoruba were used as an outgroup [51]. We also used this test to identify admixture within a target population, from two ancestral source populations [52]. For Nepalese subgroups that we identified to be admixed, we used the d-statistic to identify the direction of gene flow [53]. The HGDP Yoruba Africans were used as an outgroup population and the Tibetans and Indians were assigned as the ancestral source populations to investigate gene flow from north and south of the Himalaya. To then quantify admixture proportions the F4 ratio estimation was implemented [20, 52]. We used the HGDP Africans as the population outgroup. This method is similar to the d-statistic but assumes the correct historical phylogeny for the given populations [20, 52]. To support our proposed historical population model we used treemix to verify the historical population relationships. Finally we dated the time since the admixture event in our admixed populations using the rate of exponential decay of admixture LD computed by the package Rolloff, again using Tibetans and ANI Indians as reference populations for admixture, we used a generation time of 25 years [54, 55].

### Treemix

To model gene flow patterns between populations north and south of the Himalaya into Nepal we used the software tool Treemix (V 1.12) [56]. This was performed on our full dataset of (53,522 SNPs) using populations from Tibet, China and India and our Nepalese populations including the Sherpa. We used a standard protocol as outlined in the software tool. We found the best fit to represent the data was to allow three migration events.

### The fixation index

Weir and Cockerham’s pairwise F_st_ was used to measure population differentiation [57]. F_st_ calculations were performed on the “thinned” datasets.

### Runs of homozygosity

We performed ‘runs of homozygosity’ (ROH) analysis in PLINK, applying the following parameters: a cross-genome sliding window of 5 Mb, allowing 1 heterozygous and 5 missing calls through a ROH. A density of SNP coverage within the ROH was set as no more than 1 SNP per 50 kb. Thresholds for accepting ROH, were set to runs of at least 100 consecutive homozygous SNPs spanning lengths of 1, 5, 10 and 16 Mb of the genome. The total mean ROH length across the genome was then calculated per population and plotted using Matlab. All ROH analysis was performed on the “full” datasets, 201,573 SNPs and 637,670 SNPs for the Sherpa and Nepalese.

### Testing for consanguineous unions

We tested for consanguineous unions by comparing identity-by-descent (IBD) runs between pairs of individuals within a population, to IBD runs within individuals (i.e., ROH) of the same population. Where consanguineous unions are common in a population, one expects longer ROH within, compared to IBD between individuals of that population. We used default parameters in PLINK (v1.07) to calculate IBD segments between individuals on a “full” dataset, and ROH within an individual was calculated as described above. We specified a segment-length threshold of >16 Mb for both ROH and IBD, as recent consanguinity would generate runs above this threshold. Where pairs of individuals did not share segments of IBD satisfying the threshold we assigned a value of zero. IBD segment values were summed for all autosomes between each pair of individuals. The summed IBD segments between pairs were compared to the ROH within individuals using a two-tailed unequal variance *T*-test.

## Additional files

Additional file 1: Tables.doc. This contains seven supplemental tables. (DOC 261 kb)
Additional file 2: Figures.doc. This contains four supplemental figures. (DOC 382 kb)
Additional file 3: Cohort Description.doc. Supplemental cohort description includes cohort description of Nepalese ethnic groups. (DOC 34 kb)

## Abbreviations

AAA: Ancestral Austro-Asiatic
ANI: Ancestral north India
ASI: Ancestral south India
ATB: Ancestral Tibeto-Burman
HGDP: Human genome diversity project
IBD: Identity by descant
IBD: Quality control
PCA: Principal component analysis
ROH: Runs of homozygosity
