# Supplementary material for: Genetic structure in the Sherpa and neighboring Nepalese populations
Source: BMC Genomics. 2017 Jan 19;18:102. doi: 10.1186/s12864-016-3469-5 (PMC5248489; doi:10.1186/s12864-016-3469-5)
Supplement: Additional file 1: — Tables.doc. This contains seven supplemental tables. (DOC 261 kb) [file 12864_2016_3469_MOESM1_ESM.doc]

Table S1. The most common Nepalese ethnic groups in eastern Nepal

| Caste ethnicity | Eastern Mountain region | Eastern Hill region |
| --- | --- | --- |
| Total population size | 392,089 | 1,601,347 |
| Magar | 11,771 | 119,267 |
| Tamang | 32,708 | 113,693 |
| Newar | 12,159 | 70,396 |
| Rai | 44,133 | 332,878 |
| Percentage of total population | 41% | 58% |

Legend table S1. Information taken from the 2011 Nepalese census [4]. The percentage of the total population is calculated as the sum of these ethnic groups divided by the total population of the area. The 2011 Nepal census has classified Nepal into a number of regions including an Eco-Development Region – consisting of the Eastern Mountain region and the Eastern Hill region, which corresponds to the general region of our cohort sampling in Nepal.

Table S2. Cohort details

| **Country** | **Village** | **Ethnicity** | **Altitude (m)** | **long** | **latit** | **Language Family** |
| --- | --- | --- | --- | --- | --- | --- |
| Nepal | Duwakot | Newar (N) | 1369 | 85.41 | 27.69 | Tibeto-Burman |
| Nepal | Forte (F) | Sherpa | 3840 | 86.75 | 27.85 | Tibeto-Burman |
| Nepal | Kathmandu | Indo-Aryan (AY) | 1400 | 85.31 | 27.72 | Indo-European |
| Nepal | Kathmandu | Newar | 1400 | 85.31 | 27.72 | Tibeto-Burman |
| Nepal | Khumjung (Kh) | Sherpa | 3970 | 86.79 | 27.94 | Tibeto-Burman |
| Nepal | Kunde | Rai (R) | 3820 | 86.7 | 27.82 | Tibeto-Burman |
| Nepal | Kunde (Ku) | Sherpa | 3820 | 86.7 | 27.82 | Tibeto-Burman |
| Nepal | Lukla | Magar (M) | 2800 | 86.72 | 27.68 | Tibeto-Burman |
| Nepal | Lukla | Rai | 2800 | 86.72 | 27.68 | Tibeto-Burman |
| Nepal | Lukla | Tamang (T) | 2800 | 86.72 | 27.68 | Tibeto-Burman |
| Nepal | Lukla (L) | Sherpa | 2800 | 86.72 | 27.68 | Tibeto-Burman |
| Nepal | Namche (Na) | Sherpa | 3446 | 86.71 | 27.8 | Tibeto-Burman |
| Nepal | Solukhumbu | Rai | 3000 | 86.67 | 27.7 | Tibeto-Burman |
| Nepal | Solukhumbu (S) | Sherpa | 3000 | 86.67 | 27.7 | Tibeto-Burman |
| Nepal | Solukhumbu | Tamang | 3000 | 86.67 | 27.7 | Tibeto-Burman |
| Nepal | Thame (Th) | Sherpa | 3800 | 86.65 | 27.83 | Tibeto-Burman |
| Tibet | Lhasa (Lh) | Tibet | 3700 | 91.17 | 29.65 | Tibeto-Burman |
| Tibet | Tuo Tuo river (TTR) | Tibet | 4500 | 104.919 | 29.92 | Tibeto-Burman |
| Tibet | UK | Tibet | na | na | na | Tibeto-Burman |
| Tibet | Yunnan (Y) | Tibet | 3200-3500 | 101.69 | 24.27 | Tibeto-Burman |
| China | Beijing | Han | 44 | 116.413 | 39.92 | Sino-Tibetan |
| India | Khatri (KSH)^1^ | North India | na | na | na | Indo-European |
| India | Gujarati (GBR) ^1^ | Northwest India | na | na | na | Indo-European |
| India | West Bengal Brahmin (WBR)^1^ | East India | na | na | na | Indo-European |
| India | Maratha (MRT)^1^ | West India | na | na | na | Indo-European |
| India | Iyer (IYR)^1^ | South India | na | na | na | Dravidian |
| India | Pallan (PLN)^1^ | South India | na | na | na | Dravidian |
| India | Kadar (KAD)^2^ | South India | na | na | na | Dravidian |
| India | Irula (IRL)^2^ | South India | na | na | na | Dravidian |
| India | Paniya (PNY)^2^ | South India | na | na | na | Dravidian |
| India | Gond (GND)^3^ | Central India | na | na | na | Dravidian/Austro-Asiatic |
| India | Ho^3^ | Central and East India | na | na | na | Austro-Asiatic |
| India | Santal (SAN)^3^ | Central and East India | na | na | na | Austro-Asiatic |
| India | Korwa (KOR)^3^ | Central India | na | na | na | Austro-Asiatic |
| India | Bihor (BIR)^3^ | Central India | na | 86.07 | 25.76 | Indo-European |
| India | Manipuri Brahmin (MPB)^4^ | Northeast India | na | na | na | Tibeto-Burman |
| India | Tharu (THR)^4^ | North India | na | na | na | Indo-European |
| India | Tripuri (TRI)^4^ | Northeast India | na | na | na | Tibeto-Burman |
| India | Jamatia (JAM)^4^ | Northeast India | na | na | na | Tibeto-Burman |
| Kazakhstan | Alga | Kazakhs | 99 | 58.58 | 49.78 | Turkic |
| Kazakhstan | Almaty | Kazakhs | 900 | 76.85 | 43.22 | Turkic |
| Kyrgyzstan | Kyrgyzstan | Kyrghz | 800 | 74.74 | 41.53 | Turkic |
| Tajikistan | Kalaikhum | Tajiks | 1200 | 70.78 | 38.46 | Indo-European |
| Tajikistan | Khorog | Tajiks | 2042 | 71.56 | 37.47 | Indo-European |
| Tajikistan | Rushan | Tajiks | 6083 | 71.56 | 37.94 | Indo-European |
| Tajikistan | Shing | Tajiks | 1675 | 67.8 | 39.28 | Indo-European |
| Tajikistan | Zeravshan | Tajiks | 5489 | 68.53 | 39.18 | Indo-European |
| Uzbekistan | Bukhara | Uzbek | 225 | 64.45 | 39.76 | Turkic |
| Uzbekistan | Karshi | Uzbek | 374 | 65.78 | 38.86 | Turkic |
| Uzbekistan | Tashkent | Uzbek | 500 | 69.24 | 41.29 | Turkic |
|  |  |  |  |  |  |  |

Legend table S2. Details of our Himalayan cohort are described in the above table. The country, ethnic group, and village were documented. The longitude (long), latitude (lat) and altitude were estimated using the village name in Google maps. Each of the Indian ethnic groups are assigned to an ancestral group as defined by Basu et al., 2016; ^1^ANI, ^2^ASI, ^3^AAA and ^4^ATB. The language was recorded as the main language family for that country.

Table S3. Outgroup F_3_-statistics calculated between Himalayan populations

| Pop1 | Pop2 | F3 | std.err | Z | SNPs |
| --- | --- | --- | --- | --- | --- |
| Sherpa | Nepal | 0.176 | 0.001445 | 121.624 | 183952 |
| Sherpa | Tibet | 0.196 | 0.001966 | 99.768 | 55615 |
| Sherpa | Han | 0.192 | 0.001591 | 120.66 | 181126 |
| Sherpa | India | 0.175 | 0.001417 | 123.808 | 176473 |
| Sherpa | Pamir | 0.155 | 0.001395 | 110.876 | 164122 |
| Nepal | Tibet | 0.176 | 0.001763 | 99.643 | 56958 |
| Nepal | Han | 0.174 | 0.001358 | 128.364 | 280016 |
| Nepal | India | * | * | * | * |
| Nepal | Pamir | 0.151 | 0.001306 | 115.851 | 163540 |
| Tibet | Han | 0.197 | 0.001929 | 102.339 | 56929 |
| Tibet | India | 0.175 | 0.001774 | 98.398 | 49662 |
| Tibet | Pamir | 0.156 | 0.001691 | 91.721 | 50336 |
| Han | India | 0.177 | 0.001382 | 128.28 | 255425 |
| Han | Pamir | 0.157 | 0.00139 | 112.73 | 163320 |
| India | Pamir | 0.151 | 0.001264 | 119.454 | 157688 |

Legend table S3. Outgroup 3-population Fst was calculated using the HGDP Yoruba as an outgroup. Larger outgroup F3 scores indicate populations are more closely related. The most closely related populations across the Himalayan region are the Han and Tibetans (0.197). This degree of genetic variance is also similar for the Sherpa-Han and Sherpa- Tibet (0.192 and 0.196). The Pamir populations appear to have the most genetic variance from other Himalayan populations with f3 scores of 0.15.

Table S4. Finescale analysis of genetic variance between the Nepalese and Indian ethnic groups

| **Source** | **Source** | **Target** | **f_3** | **std.err** | **Z** | **SNPs** |
| --- | --- | --- | --- | --- | --- | --- |
| West_Bengal | Rai | Yoruba | 0.143 | 0.001238 | 115.189 | 273329 |
| West_Bengal | Indo-Aryan | Yoruba | 0.150 | 0.0012 | 124.967 | 273505 |
| West_Bengal | Magar | Yoruba | 0.143 | 0.001233 | 115.858 | 273020 |
| West_Bengal | Newar | Yoruba | 0.146 | 0.001165 | 125.42 | 273722 |
| West_Bengal | Tamang | Yoruba | 0.144 | 0.001216 | 118.418 | 273670 |
| Birhor | Newar | Yoruba | 0.153 | 0.001257 | 122.11 | 273192 |
| Birhor | Indo-Aryan | Yoruba | 0.146 | 0.001241 | 117.643 | 273002 |
| Birhor | Rai | Yoruba | 0.158 | 0.001358 | 116.274 | 272935 |
| Birhor | Magar | Yoruba | 0.157 | 0.001379 | 113.893 | 272683 |
| Birhor | Tamang | Yoruba | 0.156 | 0.001323 | 118.089 | 273199 |
| Gond | Rai | Yoruba | 0.153 | 0.001288 | 118.872 | 273257 |
| Gond | Indo-Aryan | Yoruba | 0.147 | 0.001206 | 122.263 | 273374 |
| Gond | Magar | Yoruba | 0.152 | 0.001312 | 116.209 | 272963 |
| Gond | Tamang | Yoruba | 0.152 | 0.001267 | 120.251 | 273561 |
| Gond | Newar | Yoruba | 0.152 | 0.001205 | 125.876 | 273610 |
| Gujrati | Rai | Yoruba | 0.139 | 0.001226 | 113.579 | 273346 |
| Gujrati | Indo-Aryan | Yoruba | 0.150 | 0.001185 | 126.642 | 273535 |
| Gujrati | Newar | Yoruba | 0.144 | 0.00116 | 124.266 | 273750 |
| Gujrati | Magar | Yoruba | 0.140 | 0.001234 | 113.155 | 273050 |
| Gujrati | Tamang | Yoruba | 0.141 | 0.00121 | 116.627 | 273684 |
| Jamatia | Rai | Yoruba | 0.184 | 0.001434 | 128.431 | 273307 |
| Jamatia | Indo-Aryan | Yoruba | 0.145 | 0.001233 | 117.747 | 273265 |
| Jamatia | Magar | Yoruba | 0.181 | 0.001443 | 125.369 | 273001 |
| Jamatia | Tamang | Yoruba | 0.177 | 0.001388 | 127.702 | 273555 |
| Jamatia | Newar | Yoruba | 0.166 | 0.001303 | 127.506 | 273526 |
| Kadar | Rai | Yoruba | 0.149 | 0.001267 | 117.814 | 273196 |
| Kadar | Indo-Aryan | Yoruba | 0.147 | 0.00119 | 123.245 | 273329 |
| Kadar | Magar | Yoruba | 0.149 | 0.001277 | 116.644 | 272904 |
| Kadar | Tamang | Yoruba | 0.149 | 0.001241 | 120.338 | 273497 |
| Kadar | Newar | Yoruba | 0.149 | 0.001188 | 125.522 | 273526 |
| Khatri | Rai | Yoruba | 0.137 | 0.001231 | 111.71 | 273307 |
| Khatri | Indo-Aryan | Yoruba | 0.150 | 0.001199 | 124.937 | 273499 |
| Khatri | Magar | Yoruba | 0.138 | 0.001244 | 110.905 | 273010 |
| Khatri | Tamang | Yoruba | 0.140 | 0.001222 | 114.273 | 273648 |
| Khatri | Newar | Yoruba | 0.143 | 0.001167 | 122.22 | 273700 |
| Korwa | Rai | Yoruba | 0.158 | 0.001313 | 120.461 | 273138 |
| Korwa | Indo-Aryan | Yoruba | 0.147 | 0.001223 | 120.232 | 273210 |
| Korwa | Magar | Yoruba | 0.157 | 0.00133 | 118.318 | 272855 |
| Korwa | Tamang | Yoruba | 0.157 | 0.001293 | 121.253 | 273421 |
| Korwa | Newar | Yoruba | 0.154 | 0.001221 | 126.295 | 273434 |
| Manipuri | Rai | Yoruba | 0.171 | 0.001373 | 124.591 | 273372 |
| Manipuri | Indo-Aryan | Yoruba | 0.148 | 0.001218 | 121.242 | 273472 |
| Manipuri | Magar | Yoruba | 0.169 | 0.001386 | 121.772 | 273057 |
| Manipuri | Tamang | Yoruba | 0.167 | 0.001348 | 123.598 | 273685 |
| Manipuri | Newar | Yoruba | 0.160 | 0.001259 | 127.309 | 273715 |
| Martha | Indo-Aryan | Yoruba | 0.150 | 0.001213 | 123.491 | 272982 |
| Martha | Magar | Yoruba | 0.145 | 0.001273 | 113.649 | 272593 |
| Martha | Magar | Yoruba | 0.145 | 0.001273 | 113.649 | 272593 |
| Martha | Newar | Yoruba | 0.148 | 0.001189 | 124.195 | 273126 |
| Paniya | Rai | Yoruba | 0.151 | 0.001291 | 116.704 | 272952 |
| Paniya | Indo-Aryan | Yoruba | 0.147 | 0.001207 | 121.823 | 273065 |
| Paniya | Magar | Yoruba | 0.150 | 0.001306 | 115.151 | 272685 |
| Paniya | Tamang | Yoruba | 0.151 | 0.001283 | 117.346 | 273208 |
| Paniya | Tamang | Yoruba | 0.151 | 0.001283 | 117.346 | 273208 |
| Paniya | Newar | Yoruba | 0.150 | 0.001211 | 123.778 | 273232 |
| Santal | Rai | Yoruba | 0.157 | 0.0013 | 121.078 | 273263 |
| Santal | Indo-Aryan | Yoruba | 0.148 | 0.001196 | 123.416 | 273376 |
| Santal | Magar | Yoruba | 0.157 | 0.00132 | 118.808 | 272974 |
| Santal | Tamang | Yoruba | 0.156 | 0.001281 | 121.956 | 273567 |
| Santal | Newar | Yoruba | 0.154 | 0.001215 | 126.708 | 273605 |
| Tharu | Rai | Yoruba | 0.177 | 0.001366 | 129.389 | 273319 |
| Tharu | Indo-Aryan | Yoruba | 0.147 | 0.001218 | 120.756 | 273373 |
| Tharu | Magar | Yoruba | 0.175 | 0.001384 | 126.079 | 273028 |
| Tharu | Tamang | Yoruba | 0.172 | 0.001336 | 128.726 | 273604 |
| Tharu | Newar | Yoruba | 0.163 | 0.001262 | 129.369 | 273628 |
| Tripuri | Rai | Yoruba | 0.184 | 0.001429 | 128.64 | 273333 |
| Tripuri | Indo-Aryan | Yoruba | 0.145 | 0.001227 | 118.383 | 273315 |
| Tripuri | Magar | Yoruba | 0.180 | 0.001443 | 125.025 | 273014 |
| Tripuri | Tamang | Yoruba | 0.177 | 0.001386 | 127.725 | 273598 |
| Tripuri | Newar | Yoruba | 0.166 | 0.001305 | 127.317 | 273583 |
| Pallan | Rai | Yoruba | 0.146 | 0.001262 | 115.765 | 273307 |
| Pallan | Indo-Aryan | Yoruba | 0.149 | 0.001193 | 124.936 | 273485 |
| Pallan | Magar | Yoruba | 0.146 | 0.001263 | 115.485 | 273014 |
| Pallan | Tamang | Yoruba | 0.147 | 0.001241 | 118.312 | 273635 |
| Pallan | Newar | Yoruba | 0.148 | 0.001177 | 125.879 | 273700 |

Legend Table S4. Outgroup 3-population Fst was calculated using the HGDP Yoruba as an outgroup. Larger outgroup F3 scores indicate populations are more closely related. The Nepalese appear most closely related to ATB Indians (see table S2) with F3 scores >0.18, with the exception of the Nepalese Indo-Aryan.

Table S5. Weir and Cockerham’s pairwise F_st_ between Nepalese ethnic groups and the Sherpa

| Nepal Ethnic Groups | Sherpa |
| --- | --- |
| Rai | 0.0142 |
| Magar | 0.0178 |
| Tamang | 0.0194 |
| Newar | 0.0300 |
| Indo-Aryan | 0.0560 |

Legend table S5. The genetic distance was measured between each of the seven Nepalese ethnic groups and the Sherpa residing in close proximity. Smaller F_st_ values indicate populations are more closely related. Each of the Nepalese ethnic groups have distinguishable genetic variance with the Sherpa.

Table S6. Testing for significant admixture events across the Himalaya by F3

| **A** | **B, C** | **f3** | **std.err** | **z score** |
| --- | --- | --- | --- | --- |
| Nepal | ANI, Tibet | -0.0044972 | 7.41E-05 | -60.73 |
| Nepal | Tibet, ASI | -0.0036227 | 7.28E-05 | -49.73 |
| Nepal | Tibet, AAA | -0.0027868 | 6.65E-05 | -41.89 |
| Nepal | ATB, Tibet | 0.0003492 | 3.44E-05 | 10.14 |
| Sherpa | Han, Tibet | 0.0045379 | 9.66E-05 | 46.95 |
| Sherpa | Han, Nepal | 0.0048976 | 1.20E-04 | 40.68 |
| Sherpa | Han, ANI | 0.005025 | 1.51E-04 | 33.29 |
| Sherpa | Han, AAA | 0.0059026 | 1.60E-04 | 36.92 |
| Sherpa | Han, ASI | 0.0051825 | 1.57E-04 | 32.97 |
| Sherpa | Han, ATB | 0.0059898 | 1.32E-04 | 45.54 |
| Sherpa | Tibet, Nepal | 0.0037028 | 8.06E-05 | 45.92 |
| Sherpa | Tibet, ANI | 0.0035278 | 1.02E-04 | 34.48 |
| Sherpa | Tibet, AAA | 0.003725 | 1.04E-04 | 35.98 |
| Sherpa | Tibet, ASI | 0.0036347 | 1.06E-04 | 34.27 |
| Sherpa | Tibet, ATB | 0.0040108 | 8.18E-05 | 49.03 |
| Tibet | Han, Sherpa | -0.000252839 | 4.51E-05 | -5.60768 |

Legend Table S6. Admixture was tested for in the Nepalese (A) using two ancestral source populations (B and C), the Tibetans from north of the Himalaya and each of the main Indian ancestral groups for south of the Himalaya (f3 (A; B, C)). The Sherpa were tested for admixture using a combination of Tibetan, Han and Indian source populations. Sherpa and Han ancestry in the Tibetans was confirmed. For the 3-population test a negative f3 value and Z<-5 was considered to be a significant signal of admixture.

**Table S7. D-statistic analysis to infer gene flow direction in Nepalese subgroups**

| **Pop1** | **Pop2** | **Pop3** | **Pop4** | **D-statistic** | **Z** |
| --- | --- | --- | --- | --- | --- |
| ANI | Newar | Tibet | Yoruba | -0.0583 | -47.269 |
| ANI | Magar | Tibet | Yoruba | -0.0943 | -54.118 |
| ANI | Tamang | Tibet | Yoruba | -0.0859 | -57.489 |
| ANI | Rai | Tibet | Yoruba | -0.1013 | -58.907 |
| ANI | Indo-Aryan | Tibet | Yoruba | -0.0086 | -8.656 |
| Han | Tibet | Newar | Yoruba | -0.0025 | -3.683 |
| Han | Tibet | Magar | Yoruba | -0.0035 | -4.613 |
| Han | Tibet | Tamang | Yoruba | -0.0051 | -7.139 |
| Han | Tibet | Rai | Yoruba | -0.005 | -6.511 |
| Han | Tibet | Indo-Aryan | Yoruba | -0.0025 | -3.439 |

Legend Table S7. The D-statistic test was performed using two source populations, Pop1 and Pop3 and a third potentially admixed population Pop2 (D(((P_1_, P_2_), P_3_), P_4_)). The Yoruba African HGDP were used as an outgroup population Pop4. A negative D-statistic indicates that gene flow occurred between Pop2 and Pop3. A Z score < -5 indicates significance.
