# Supplementary material for: Genetic structure in the Sherpa and neighboring Nepalese populations
Source: BMC Genomics. 2017 Jan 19;18:102. doi: 10.1186/s12864-016-3469-5 (PMC5248489; doi:10.1186/s12864-016-3469-5)
Supplement: Additional file 2: — Figures.doc. This contains four supplemental figures. (DOC 382 kb) [file 12864_2016_3469_MOESM2_ESM.doc]

Figure S1. Detailed population structure of the ‘northern Himalayan’ cluster

A
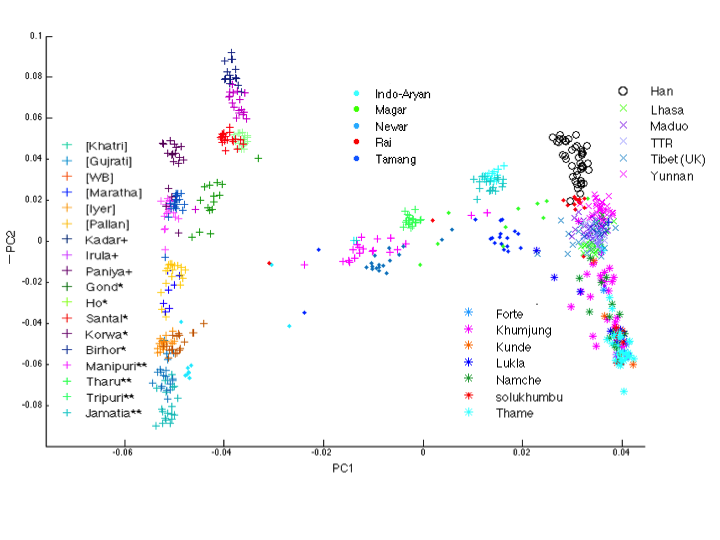


B


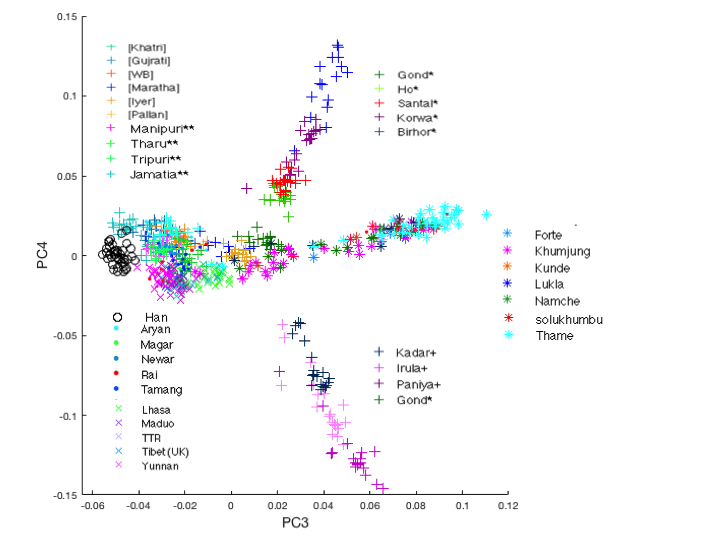


Legend Fig S1. These principal component analyses focus on the ‘northern Himalayan’ cluster identified in figure 1. The population labels are detailed in figure 1. A) PC1 separates the northern and southern Himalayan populations, and PC2 separates the genetic variation within each of these clusters. B) PC3 separates the Sherpa population from their surrounding Himalayan populations and PC4 separates the AAA* Indians and ASI+ Indian populations. The Sherpa appear as a distinct isolated Himalayan population.

Figure S2. Fine-scaled population structure of eight Nepalese Sherpa villages mirrors the geographical location


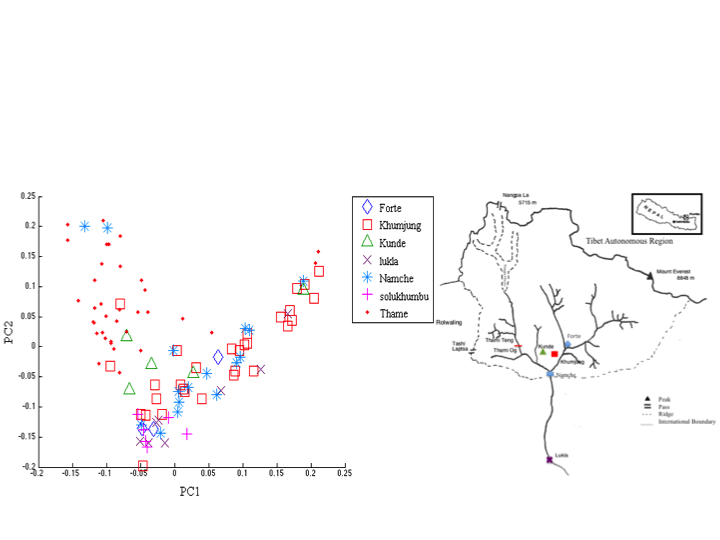


Legend Fig S2. The Principle Component Analysis reflects the geographical locations of these Nepalese villages, indicating that natural geographical boundaries are likely playing a role in gene flow (Map adapted from 52). The villages of Lukla, Namche, Khumjung, Kunde, and Forte are ‘major’ villages along the main trekking route to Mt. Everest base camp and this pathway may be responsible for the clustering of these village on the PCA. PC1 and PC2 identify individuals from Thame as a subpopulation of the Sherpa.

Figure S3. A phylogeny explaining the f4 ratio estimation


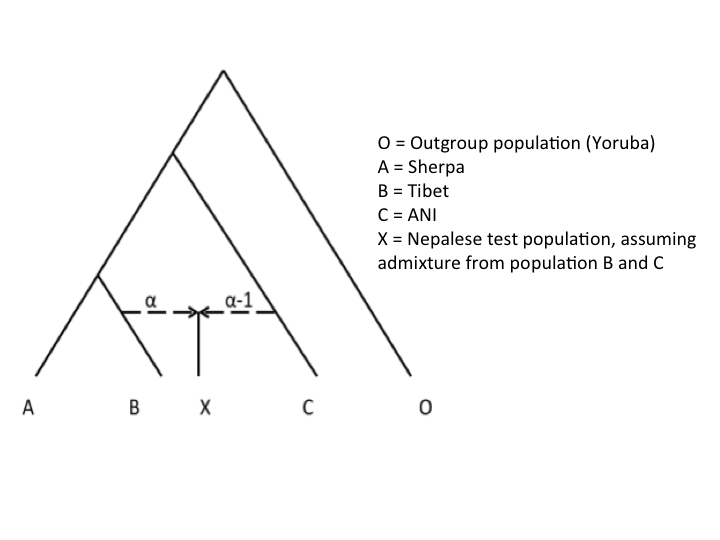


Legend Fig S3. We estimated the admixture proportions in each of our Nepalese populations ‘X’ under the assumption we have the correct historical model. The estimate of α is calculated as
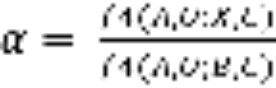
. For detailed information on f4 ratio estimation refer to (20, 46).

Fig S4. Levels of homozygosity in Himalayan populations


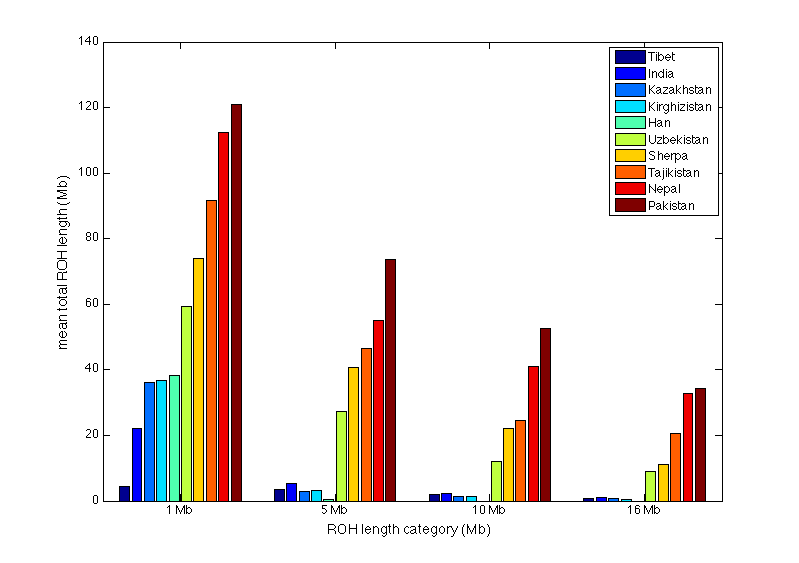


Legend Fig S4. Runs of homozygosity (ROH) were plotted on a bar graph for our Himalayan cohorts. The x-axis represents the thresholds set for defining a ROH (≥1 Mb, ≥5 Mb, ≥10 Mb and ≥ 16 Mb). The y-axis is the mean total ROH length calculated for that population. We identify particularly high levels of ROH ≥ 16 Mb in the general Nepalese population also, followed by Tajikistan and the Sherpa.

Figure S5. Investigating consanguinity in the Sherpa and Nepalese


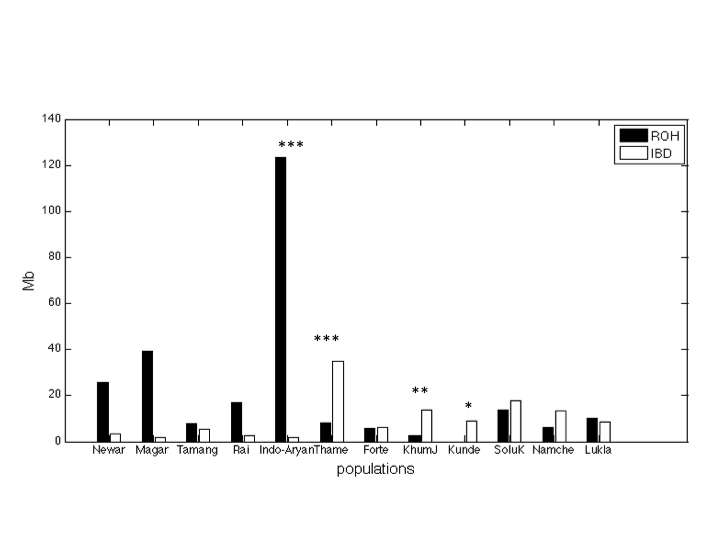


Legend Fig S5. ROH16 and IBD16 averages were calculated for each of the Sherpa and Nepalese subgroups. The x-axis indicates the subpopulation and the y-axis indicates the average segment length in Mb. Significant differences (P<0.0005) are indicated by ***, (P<0.005) by ** and (P<0.05) by *.
