# Supplementary material for: Genetic structure in the Sherpa and neighboring Nepalese populations
Source: BMC Genomics. 2017 Jan 19;18:102. doi: 10.1186/s12864-016-3469-5 (PMC5248489; doi:10.1186/s12864-016-3469-5)
Supplement: Additional file 3: — Cohort Description.doc. Supplemental cohort description includes cohort description of Nepalese ethnic groups. (DOC 34 kb) [file 12864_2016_3469_MOESM3_ESM.doc]

S1. Supplemental cohort description

According to tradition the Rai are of Mongolian or Tibetan origin and migrated to Nepal in the 7^th^ century. They are believed to be amongst the first inhabitants of the Solu region of Nepal [19]. Tradition believes their ancestral group the Kiranti (Sino-Tibetan language) entered Nepal’s eastern hills through the Barakshetra (Varahakshetra) gorge of Koshi Valley – the natural gateway into the region through the Mahabharata rage which separates the hilly region from the plains [19].

The Magar are a Himalayan tribe, believed to be of central Asian origin due to their Mongolian appearance and their ties to the Tibeto-Burman family of languages. It is also thought that their place of origin was the Kham province (Tibet) also believed to be the origin of the Sherpa. It is thought that that the Magar tribe settled in Sikkim, an Indian state bordering eastern Nepal, in the 5^th^ century and onto Nepal in the 7^th^ century. They inhabited a region between the rivers of Bheri and Matsyandhri in Nepal [19].

The Tamang have been referred to as an indigenous tribe to an area North of Kathmandu, Nepal. It is believed they were on one of the major routes into Tibet, until the British opened up the way to Lhasa through Sikkim at the beginning of the 20^th^ century [55]. The Tamang language has been classified close to Tibetan and falls within the Tibeto-Burman language branch, suggestive of a northern Himalayan ancestry [54,55]. The Tamang have also been described to have distinctive mongoloid appearance [54].

As is the general consensus for all ethnic groups in Nepal, the origin of the Newars is controversial. There have been a number of views regarding their ancestry, including, immigration from Northern Himalayan regions or possible South Indian and Austro-Asia elements [11, 57], but according to Furer-Haimendorf their existence in Kathmandu has been since pre-historic times [56]. They have been described as the indigenous population of Nepal, existing there for over 2,000 years. It is believed they may be descendants of the Kirata and Licchavis, Indian clans that arrived in Nepal in the 4^th^ century [5,6], and that the term Newar was used in general terms to describe the inhabitants of Nepal at that time [5]. Today the Newars are a dispersed group throughout Nepal and have also taken up residence in Darjeeling, Bhutan and Lhasa [22]. Traditionally the Newars were merchants involved in trade along the Silk Road, specifically between Kathmandu and Central Asia, including regions such as Lhasa, Tibet [22].

It has been suggested that the Indo-Aryan group may have initially migrated from a region of Tibet and arrived in northern India in the Vedic period (about 1750 years ago). However they continued their migration in a northeasterly direction crossing Punjab (a region of eastern Pakistan and northern India), its occupants primarily speaking the Indo-European language in the direction of the north foothills of the Himalaya [5]. The Indo-Aryan’s arrival to Nepal succeeded the rulings of the Kirats, in the Kathmandu region [21]. It has been said that the movement of the Indo-Aryan people throughout Nepal has been responsible for shaping Nepal following their establishment of new territory [58]. Systemic migration into Nepal from north eastern and southern directions has been responsible for the Nepal’s demography, and can be classified into three broad groups; Tibeto-Nepalese, Indo-Nepalese and indigenous [59].
